# No drug, no trial? Think again!

**DOI:** 10.1186/1745-6215-16-S2-P201

**Published:** 2015-11-16

**Authors:** Kirsty Sprange, Eleanor Mitchell, Mark Hull, Gill Bumphrey

**Affiliations:** 1 University Of Nottingham, Nottingham, UK; 2 University of Leeds, Leeds, UK

## 

A reliable drug supply is essential for trial success, but what happens when part way through the trial your source fails and there is no alternative supplier of the same product. This became the challenge for the seAFOod trial in 2014. seAFOod is a randomised, double-blind, placebo-controlled study to determine whether fish oil (omega-3 EPA) prevents colorectal adenomas, alone or combined with aspirin.

Whilst most sites were temporarily suspended to recruitment during 2014, a solution was sought to the EPA IMP problem. Consultation with EME, MHRA, REC and independent oversight committees was key to exploring options for continuing the trial and enabled effective decision making. Once sourcing a different product was agreed, the search was on. With no ‘quick fix’ option available, excellent communication was vital to identify an alternative source of EPA IMP and keep sites engaged and motivated to continue the trial. Once the new product was found it was important to update trial documents and arrange approvals and contracts promptly. Good time management facilitated achievement of key milestones and established realistic expectations within the research team and at sites for continuation of the trial. Finally a series of motivational re-launch events meant the trial met the funder's ‘stop/go’ recruitment target ahead of schedule.

A supportive funder, motivated Chief Investigator and trial team along with good trial management are paramount. This abstract will demonstrate the successful continuation of the seAFOod trial using an alternative equivalent IMP and present the experience and lessons learned by a Trial Management Group.

